# A simple bedside technique for managing a non-deflating Foley catheter balloon in a resource-limited setting – a case report

**DOI:** 10.1186/s12245-026-01192-3

**Published:** 2026-03-20

**Authors:** Mudacyahwa Regis, Turamyimana Faustin, Menbeu Sultan Mohammad

**Affiliations:** 1 Department of Surgery, African Health Science University, Kigali, Rwanda; 2 Emergency Medicine and Critical Care, University of Rwanda, Kigali, Rwanda

**Keywords:** Foley catheter, Non-deflating balloon, Bedside technique, Low-resource setting

## Abstract

**Background:**

Urethral catheterization is a frequently performed procedure that can sometimes result in complications such as failure to deflate a Foley catheter balloon. This can present significant challenges in managing a patient.

**Case presentation:**

We describe a case involving a 37-year-old woman diagnosed with severe cerebral malaria, eclampsia, and HELLP syndrome whose Foley catheter balloon failed to deflate despite the use of standard techniques. An easy bedside technique was used by cutting the catheter to a short length and using a spinal needle stylet to unblock the existing inflation passage, thus successfully deflating the balloon without complications or the need for anesthesia.

**Conclusion:**

This method is safe, minimally invasive, cost-effective, and particularly valuable in low-resource environments.

## Introduction

Urethral catheterization is a routine, sterile procedure used to access the bladder via the urethra for drainage. It must be performed with proper precautions, care, and adherence to guidelines, as complications can be serious and life-threatening [1]. It is the most common retrograde procedure performed on the urinary tract and is routinely done for both hospital and community patients [2]. It is estimated that 15% to 20% of patients have a Foley catheter at some point during their hospital stay [2]. 

Urethral catheters can be used for both short-term and long-term applications, depending on the specific indication, and are associated with various issues and complications. Complications related to catheters are frequently encountered, particularly among patients with long-term urethral catheterization [3]. These issues include urinary tract infections, bladder discomfort and pain, bleeding, leakage and blockage of the catheter, and, though infrequently, catheter retention. One of the primary causes of difficulty in removing a Foley catheter is a non-deflating balloon, which can create an urgent urological situation [4, 5]. Several key factors contributing to the issue of non-deflating balloons include malfunctioning catheter balloon valves and channels, the use of inflation fluids like saline or water contaminated with blood that can crystallize and obstruct the balloon channel, as well as prolonged urinary catheter use [6]. 

Various techniques and strategies have been suggested to achieve balloon deflation or rupture, including cutting the inflation valve, passing a guidewire, employing chemical agents to disrupt the balloon, and performing a surgical puncture of the balloon [7]. We introduce a straightforward and safe method for mechanically puncturing the catheter balloon in female patients with retained catheters, especially in lower-middle and low-income countries.

## Case presentation

A 37-year-old (G4P2102) at 35 weeks and 2 days of gestation was transferred for critical care and respiratory support. She was diagnosed with severe cerebral malaria, eclampsia on a background of preeclampsia, acute kidney injury (creatinine 186 µmol/L), HELLP syndrome with severe thrombocytopenia (platelets 7,000/µL), severe anemia (Hb 6.3 g/dL), hyperkalemia (K⁺ 5.9 mmol/L), and metabolic acidosis (pH 7.0, HCO₃⁻ 12 mmol/L). She also presented in a comatose state and had an intrauterine fetal demise (IUFD). Her symptoms began five days prior to our admission with fever, vomiting, and joint pain, which were initially treated as bacterial sepsis. Shortly after, she developed seizures and lost consciousness before her transfer.

Upon arrival, she was critically ill with a urethral catheter in place, draining dark urine. She was hemodynamically stable, and an obstetric ultrasound showed a single intrauterine pregnancy with cephalic presentation and absent fetal cardiac activity. The diagnosis included cerebral malaria and eclampsia complicated by acute kidney injury, HELLP syndrome, severe anemia, metabolic issues, and IUFD. She was admitted to the ICU for stabilization, respiratory support, correction of metabolic issues, and blood transfusions.

After stabilization and correction of her metabolic derangements, labor was induced, and she expelled the fetus. She later improved in both her neurological and respiratory conditions and continued to receive supportive care in the ICU.

On the 20th day of her admission, the nurse in charge attempted to change the Foley catheter, but the balloon failed to deflate despite several standard attempts. The ICU doctor was immediately informed and also attempted to deflate the balloon normally, but that also failed. Even cutting the Foley catheter in half and attempting to deflate it through the inflation line directly was unsuccessful.

**Fig. 1:**
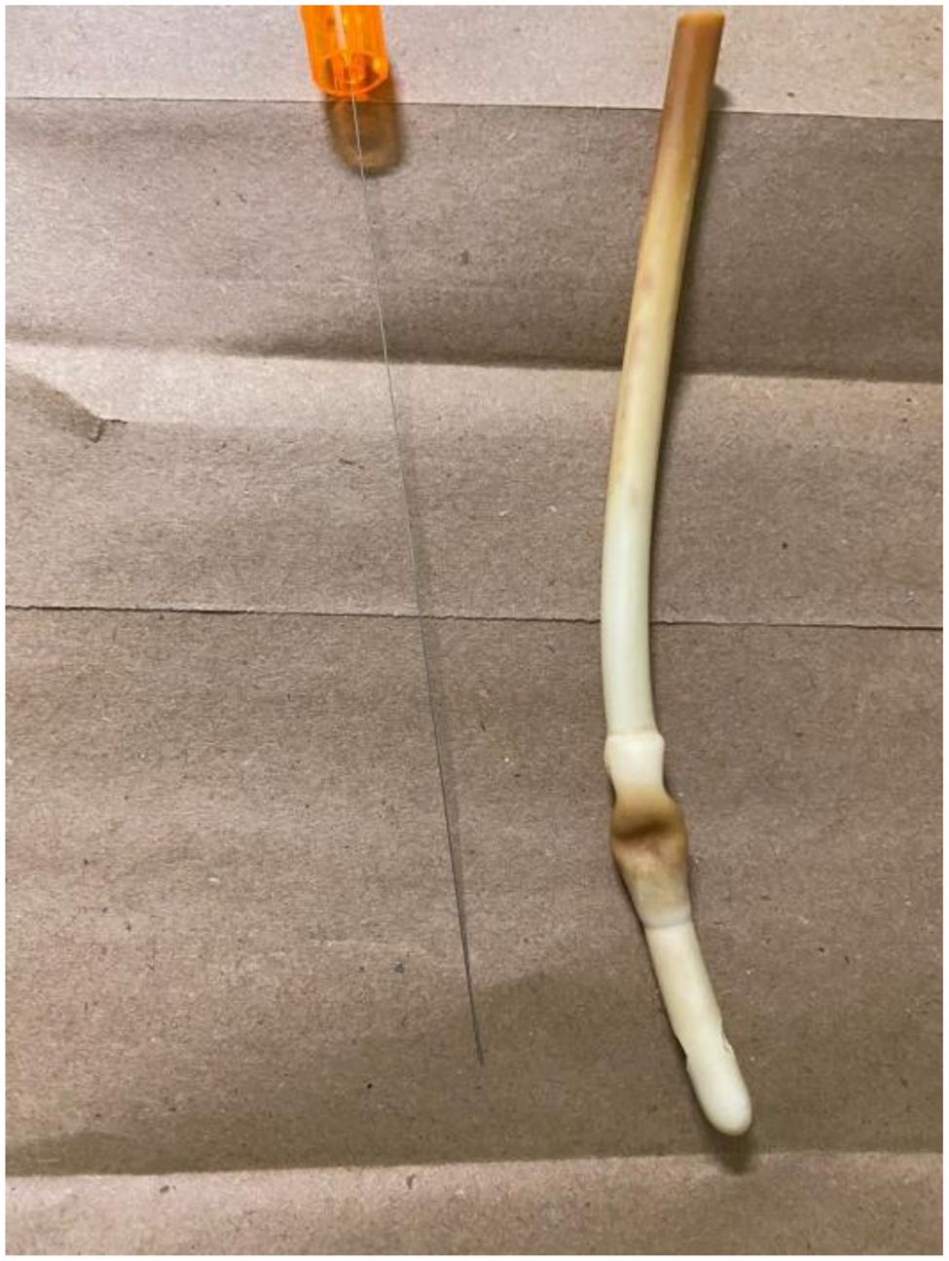
The stylet, after being used to deflate the balloon of the Foley catheter, along with the deflated balloon

**Fig. 2:**
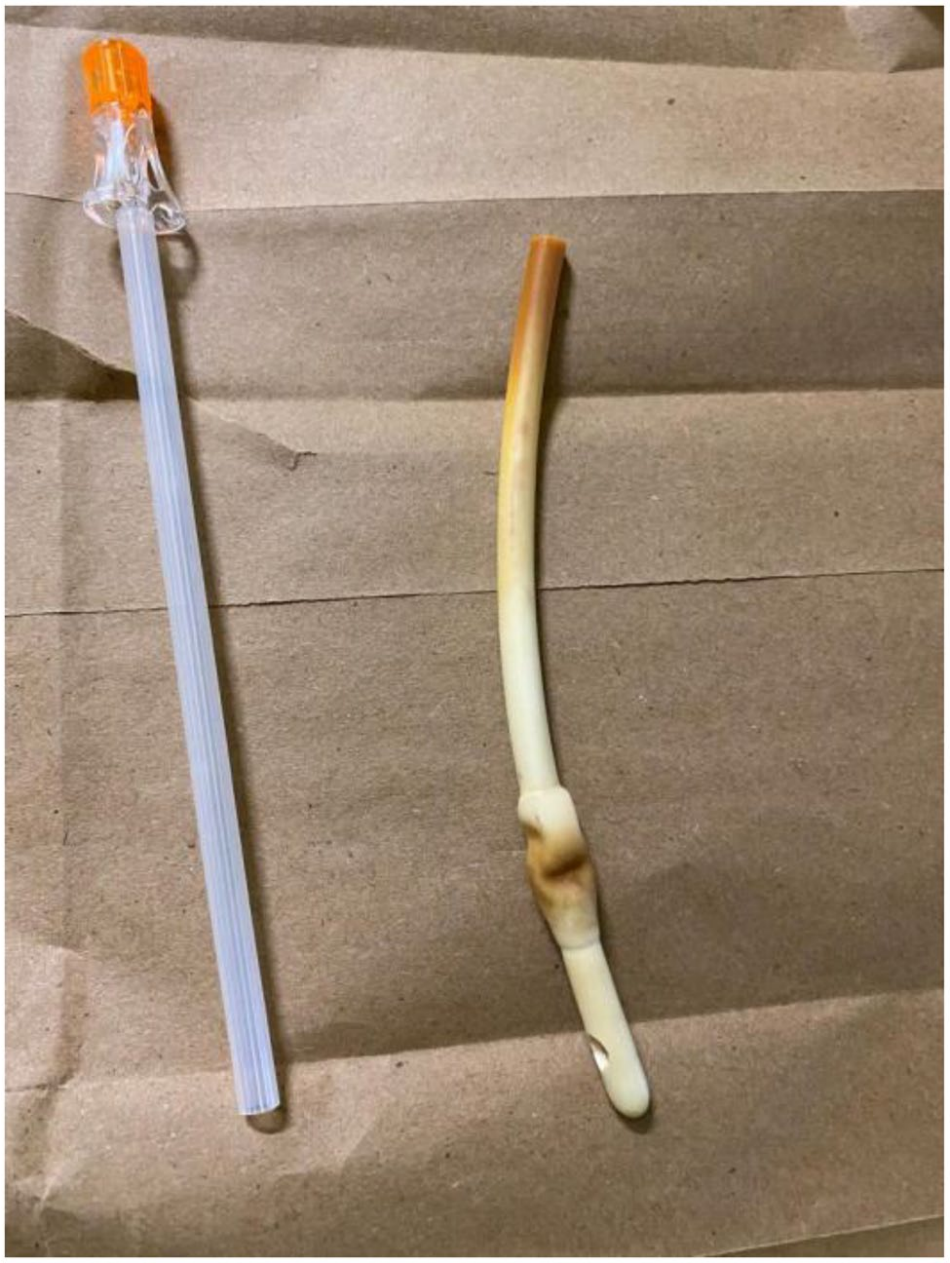
A 25-gauge spinal needle with its stylet in place, alongside the tip of a deflated Foley catheter (done with the stylet)

**Fig. 3:**
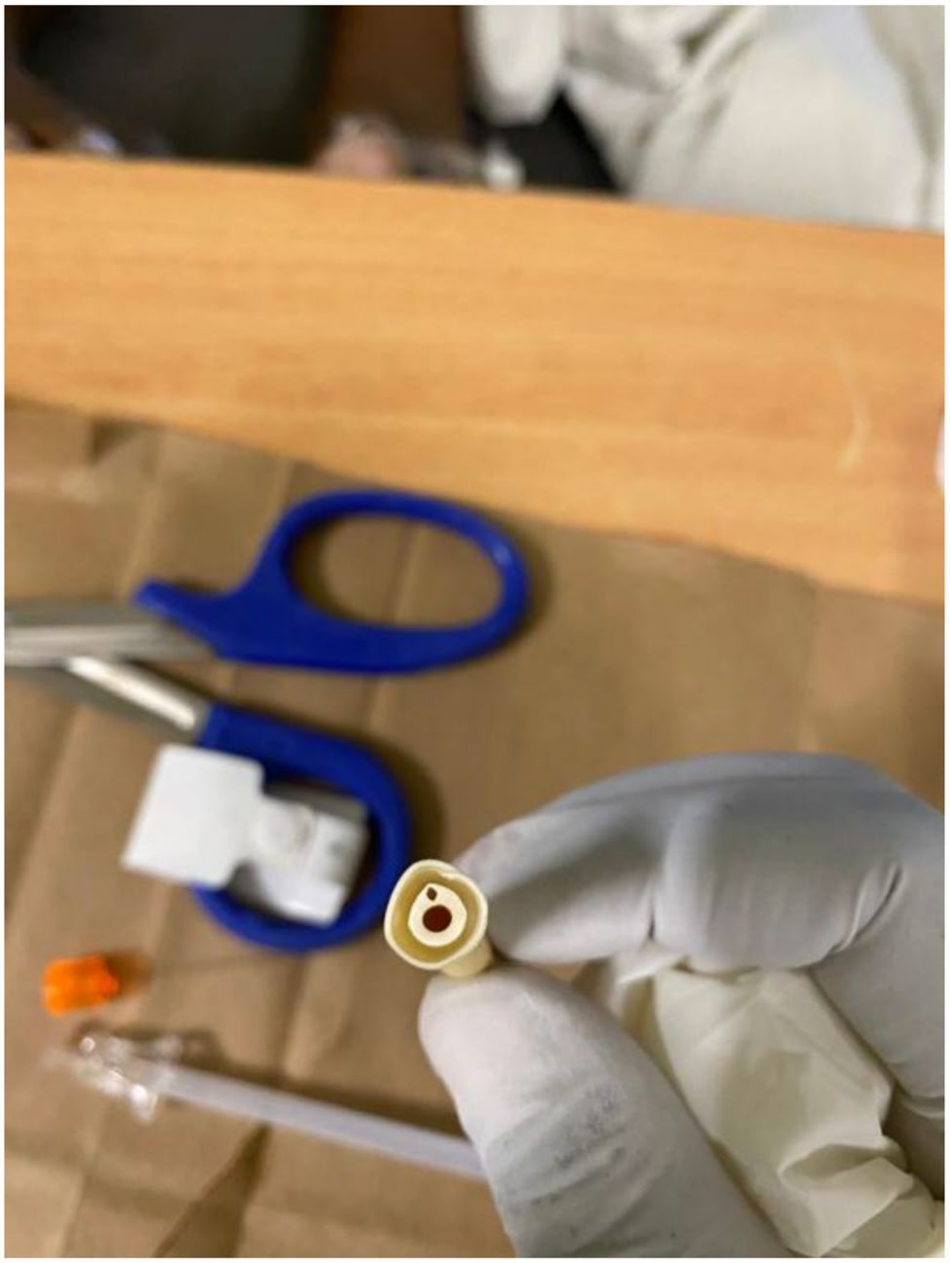
A transverse cut showing the Foley catheter duct (the larger opening) and the balloon inflation channel (the smaller opening) where it connects to the balloon

Since conventional methods were ineffective, a new bedside technique was employed. The Foley catheter was cut short using scissors, leaving approximately 10 cm of the external catheter from the vulva to the point of the cut. The stylet (Fig. 1) from a spinal needle (Fig. 2) was removed from its cannula, and the stylet itself was gently inserted into the inflation channel (Fig. 3) slowly under direct visualization to avoid bladder injury, as it was visible on the transverse cut of the Foley catheter. A slight upward pressure was applied, then the inflation fluid was drained, and the balloon deflated, allowing the catheter to be smoothly removed. The procedure was performed without anesthesia, was fast, well-tolerated, and caused no complications. A video demonstration of the technique is provided below. Written informed consent was obtained from the patient to publish this case report and any related images. A copy of the consent form can be reviewed by the Editor-in-Chief of this journal upon request.

Video 1. Demonstration of the bedside technique for deflating a non-deflating Foley catheter balloon using a spinal needle stylet. To view the demonstration of the bedside Technique: Deflating a Non-Deflating Foley Catheter Balloon, refer https://www.youtube.com/watch?v=XJgY1ebdEaU.

## Discussion

Non-deflating Foley catheter balloons, though uncommon, can pose a significant management challenge [6]. Common causes include blockage of the inflation channel by crystallized substances, valve malfunction, or balloon adhesion to the bladder wall [7, 8]. 

Standard management techniques such as cutting the inflation channel, inserting a guidewire or a small catheter into the inflation port, using chemical dissolution methods (such as mineral oil or ether), cystoscopic puncture of the balloon, or performing a suprapubic puncture under ultrasound guidance may sometimes be ineffective or require specialized tools and expertise [3]. 

The bedside technique highlighted in this case takes advantage of female urethral anatomy to provide a safe and minimally invasive solution. Given that the average female urethra measures 3–4 cm, the Foley catheter can be trimmed to a short length, allowing the needle stylet to access the balloon via the existing inflation pathway. This method avoids the creation of new tissue passages, thereby reducing discomfort and the risk of injury. To enhance safety, it is recommended that the bladder be filled with at least 120 cc of water or urine before clamping the catheter, which helps safeguard against potential bladder damage during the stylet insertion. This approach is particularly suitable for low-resource environments, enabling quick resolution without specialized equipment, invasive procedures, or anesthesia. One limitation of this report is that it describes the technique in just a single female patient. Conducting a larger case series or prospective study would yield more robust evidence regarding its reproducibility and general applicability.

## Conclusion

This case illustrates a straightforward, safe, and effective bedside technique for managing a non-deflating Foley catheter balloon when conventional methods are unsuccessful. By utilizing anatomical features and the current catheter pathway, this method minimizes patient discomfort and procedural risks, rendering it a viable option in medically constrained settings.

## Data Availability

No datasets were generated or analyzed during the current study.
